# Metabolites from *Clonostachys* Fungi and Their Biological Activities

**DOI:** 10.3390/jof6040229

**Published:** 2020-10-16

**Authors:** Peipei Han, Xuping Zhang, Dan Xu, Bowen Zhang, Daowan Lai, Ligang Zhou

**Affiliations:** Department of Plant Pathology, College of Plant Protection, China Agricultural University, Beijing 100193, China; mhanpeipei@126.com (P.H.); zhangxuping5@cau.edu.cn (X.Z.); cauxudan@163.com (D.X.); bowenzhang7@163.com (B.Z.); dwlai@cau.edu.cn (D.L.)

**Keywords:** secondary metabolites, *Clonostachys* fungi, *Bionectria*, *Gliocladium*, *Nectria*, structural diversity, biological activities

## Abstract

*Clonostachys* (teleomorph: *Bionectria*) fungi are well known to produce a variety of secondary metabolites with various biological activities to show their pharmaceutical and agrochemical applications. Up to now, at least 229 secondary metabolites, mainly including 84 nitrogen-containing metabolites, 85 polyketides, 40 terpenoids, and 20 other metabolites, have been reported. Many of these compounds exhibit biological activities, such as cytotoxic, antimicrobial, antileishmanial, antimalarial activities. This mini-review aims to summarize the diversity of the secondary metabolites as well as their occurrences in *Clonostachys* fungi and biological activities.

## 1. Introduction

The fungal genus *Clonostachys* (formerly named *Gliocladium*), teleomorph *Bionetria* (formerly named *Nectria* or *Nectriopsis*), belongs to the family Bionectriaceae of Sordariomycetes in Ascomycota [1]. The *Clonostachys* fungi are widely distributed all over the world. They are saprotrophs, destructive mycoparasites, lichenicoles, or inhabitants of recently dead trees and decaying leaves. At present, there are about 44 species in the genus *Clonostachys* [1], and among them, about 18 species have been studied for their secondary metabolites, including *B. byssicola*, *B. ochroleuca*, *B. pityrodes*, *C. candelabrum*, *C. compactiuscula*, *C. rogersoniana*, *C. rosea*, *G. roseum*, *N. coccinea*, *N. coryli*, *N. erubescens*, *N. fuckeliana*, *N. galligena*, *N. haematococca*, *N. inventa*, *N. lucida*, *N. pseudotrichia*, and *N. viridescens*.

*Clonostachys* fungi are abundant in many classes of secondary metabolites, mainly including nitrogen-containing compounds, polyketides, and terpenoids. Many metabolites exhibit biological activities, such as antimicrobial, insecticidal, nematocidal, antiparasitic, phytotoxic and cytotoxic activities. Until now, secondary metabolites of *Clonostachys* fungi and their biological activities have not been reviewed. This mini-review describes the classification, occurrences, and biological activities of the secondary metabolites from *Clonostachys* fungi.

## 2. Nitrogen-Containing Metabolites and Their Biological Activities

The nitrogen-containing metabolites from *Clonostachys* fungi mainly include linear oligopeptides, cyclopeptides, and piperazines. The nitrogen-containing metabolites, their isolated *Clonostachys* fungi and biological activities are shown in Table 1.

### 2.1. Linear Oligopeptides

**Figure 1:**
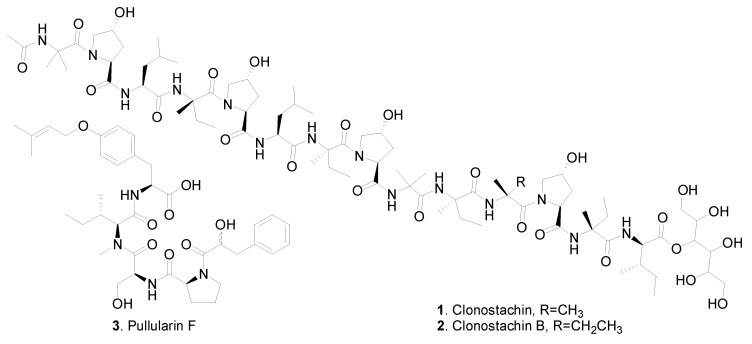
Linear oligopeptides isolated from *Clonostachys* fungi.

The oligopeptides from fungi include linear and cyclic peptides. Two linear tetradecapeptides, named clonostachin (**1**) and clonostachin B (**2**) were isolated from *Clonostachys* fungi (Figure 1). Clonostachin (**1**) was first isolated from *Clonostachys* sp. F5898, and both clonostachin (**1**) and clonostachin B (**2**) were then isolated from *Bionectria* sp. MSX 47401, and each oligopeptide contained an N-terminal acetyl group and a *C*-terminal mannitol unit [3]. Clonostachin (**1**) inhibited ADP-induced aggregation of human platelets by 80% at 150 μM [2]. Pullularin F (**3**) was isolated from the endophytic fungus *Bionectria ochroleuca* from the mangrove plant *Sonneratia caseolaris* [4].

### 2.2. Cyclopeptides

**Figure 2:**
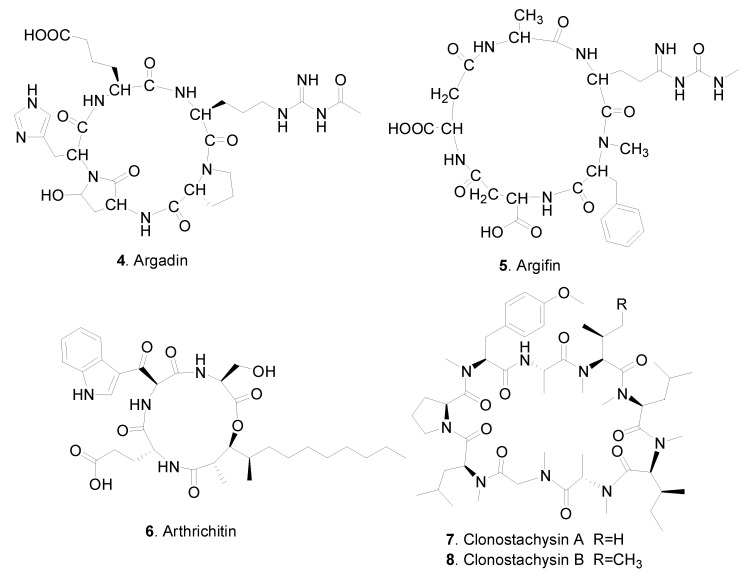

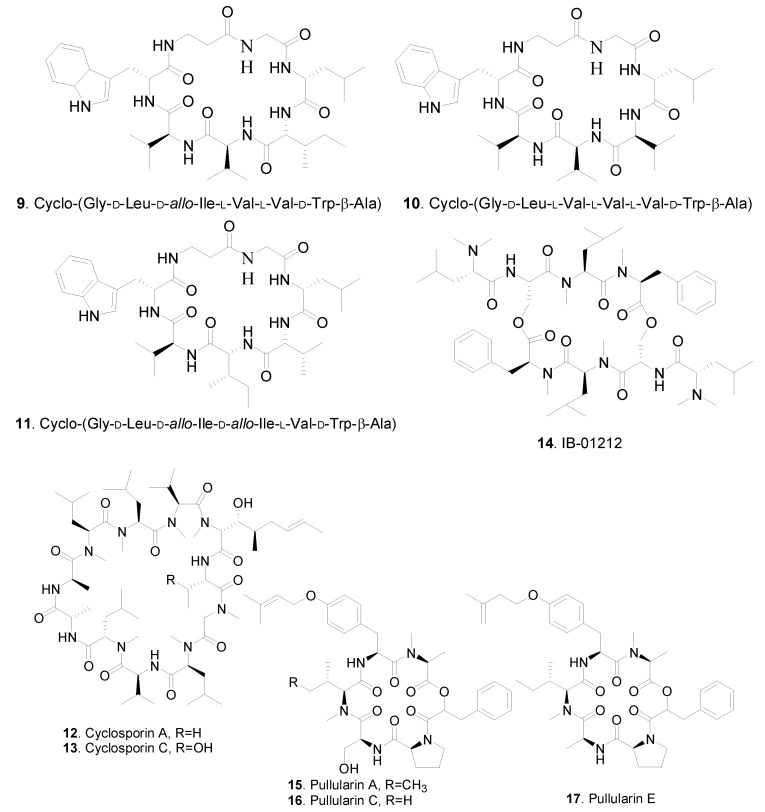
Cyclopeptides isolated from *Clonostachys* fungi.

Cyclopeptides are cyclic compounds formed mainly by the amide bonds between either proteinogenic or non-proteinogenic amino acids [13,39]. The structures of the cyclopeptides isolated from *Clonostachys* fungi are shown in Figure 2.

Argadin (**4**), a cyclic pentapeptide, was isolated from *Clonostachys* sp. FO-7314. It showed inhibitory activity against blowfly (*Lucilia cuprina*) chitinase with IC_50_ values of 150 nM at 37 °C and 3.4 nM at 20 °C, respectively [5]. Another cyclic pentapeptide, namely argifin (**5**), from *Gliocladium* sp. also exhibited inhibitory activity against blowfly chitinase [6,7].

Arthrichitin (**6**) was a cyclic tetradepsipeptide isolated from *Nectria* sp. [8]. This lipodepsipeptide was also isolated from other fungi to show inhibitory activity on the yeasts *Schizosaccharomyces pombe* and *Rhodotorula glutinis* [9].

Clonostachysins A (**7**) and B (**8**) were two cyclic nonapeptides isolated from *Clonostachys rogersoniana*. They exhibited a selectively inhibitory effect on a dinoflagellate *Prorocentrum micans* at 30 μM but had no effect on other microalgae and bacteria, even at 100 μM [10].

Three cyclic heptapeptides, named cyclo-(Gly-_D_-Leu-_D_-allo-Ile-_L_-Val-_L_-Val-_D_-Trp-β-Ala) (**9**), cyclo-(Gly-_D_-Leu-_L_-Val-_L_-Val-_L_-Val-_D_-Trp-β-Ala) (**10**), and cyclo-(Gly-_D_-Leu-_D_-allo-Ile-_D_-allo-Ile-_L_-Val-_D_-Trp-β-Ala) (**11**), were isolated from the soil-derived fungus *Clonostachys* rosea. Among them, cyclo-(Gly-_D_-Leu-_D_-allo-Ile-_L_-Val-_L_-Val-_D_-Trp-β-Ala) (**9**) exhibited significant cytotoxic activity against the L5178Y mouse lymphoma cell line with an IC_50_ value of 4.1 μM [11].

Two cyclic undecapeptides cyclosporins A (**12**) and C (**13**) were isolated from *Nectria* sp. F-4908 [12]. They showed immunosuppressive and antifungal activities [13].

IB-01212 (**14**), a cyclic hexadepsipeptide from the marine fungus *Clonostachys* sp. ESNA-A009, exhibited antitumor activity on the cell lines of LN-caP (prostate cancer), SK-BR3 (breast cancer), HT29 (colon cancer), and HeLa (cervix cancer) [14]. In addition, IB-01212 (**14**) showed antileishmanial activity [15].

Three cyclic hexadepsipeptides pullularins A (**15**), C (**16**) and E (**17**) were isolated from the endophytic fungus *Bionectria ochroleuca*. Both pullularins A (**15**) and C (**16**) showed moderate cytotoxic activity against mouse lymphoma cells [4]. Furthermore, pullularin A (**15**) from another fungus *Pullularia* sp. BCC 8613 exhibited antimalarial, antiviral and antitubercular activities [40].

### 2.3. Piperazines

**Figure 3:**
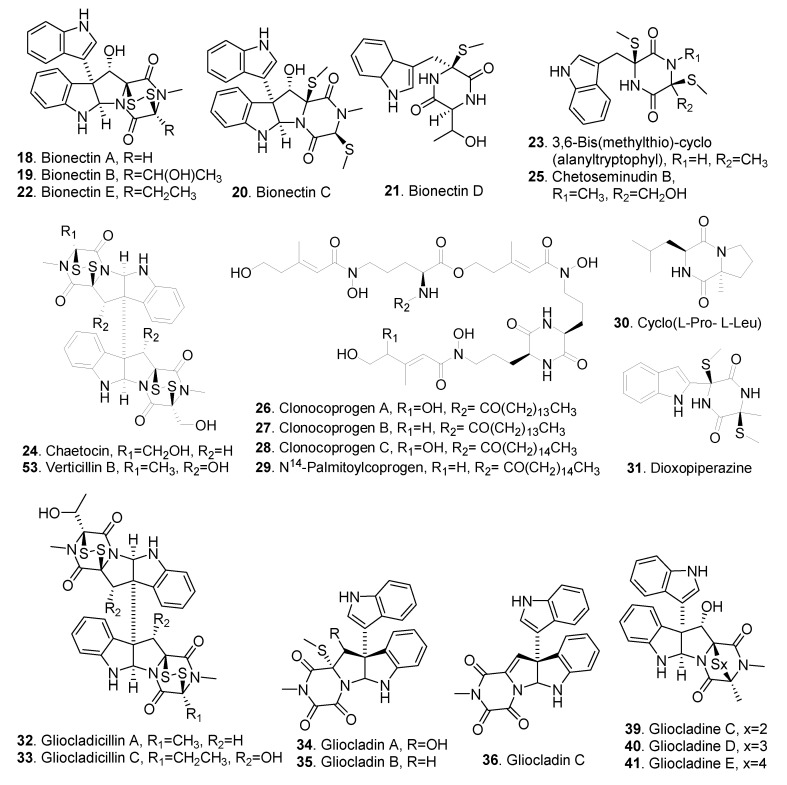

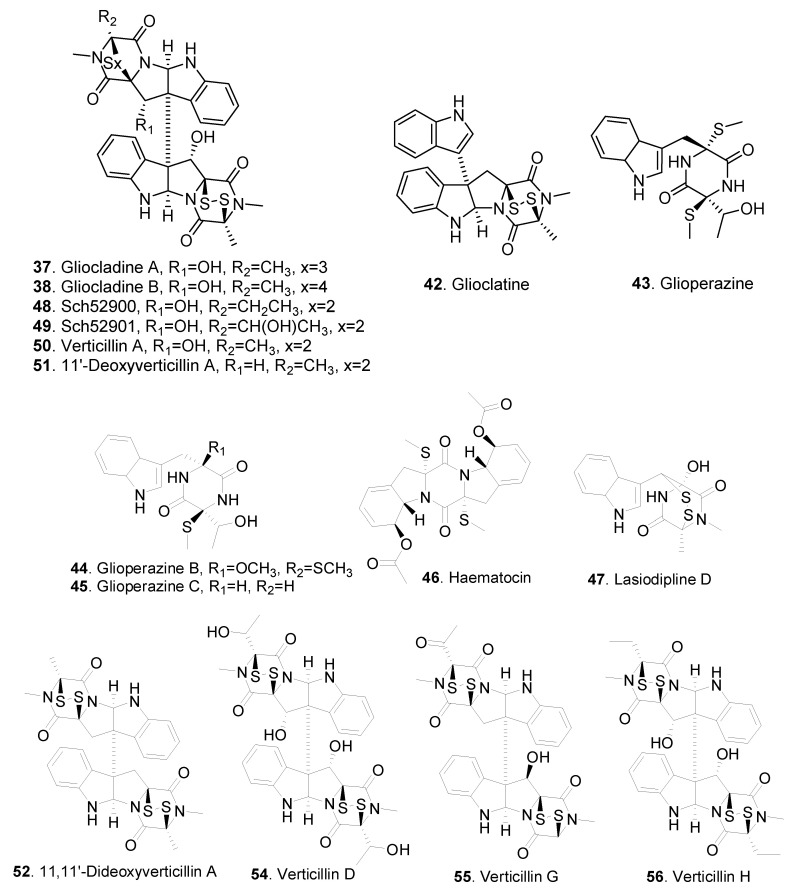
Piperazines isolated from *Clonostachys* fungi.

The piperazines (also called 2,5-diketopiperazines) are formed by the condensation of two amino acids [41]. Piperazines are the common nitrogen-containing metabolites as monomers or dimers in *Clonostachys* fungi, and most of them contain disulfide bonds. The structures of piperazines isolated from *Clonostachys* fungi are shown in Figure 3.

Bionectins A (**18**), B (**19**) and C (**20**), and verticillin D (**54**) were isolated from the liquid fermentation cultures of *Bionectria byssicola* F120. Both bionectins A (**18**) and B (**19**) exhibited antibacterial activity against *Staphylococcus aureus* including methicillin-resistant *Staphylococcus aureus* (MRSA) and quinolone-resistant *Staphylocossu aureus* (QRSA), with MIC values of 10-30 μg/mL [16]. Bionectins D (**21**) and E (**22**), cyclo (_L_-Pro-_L_-Leu) (**30**), dioxopiperazine (**31**), and gliocladicillins A (**32**) and C (**33**) were isolated from *Bionectria* sp. Y1085. Among them, bionectins D (**21**) and E (**22**), as well as gliocladicillin C (**33**) showed antibacterial activity on *Escherichia coli*, *Staphylococcus aureus* and *Salmnonella typhimurium* [17].

Four diketopiperazines: 3,6-bis(methylthio)-cyclo(alanyltryptophyl) (**23**), chaetocin (**24**), chetoseminudin B (**25**) and verticillin B (**53**) from deep water marine-derived fungus *Nectria inventa* showed trypanocidal activity on *Trypanosoma brucei* [18].

Four siderophore analogs, clonocoprogens A (**26**), B (**27**) and C (**28**) and *N*^14^-plmitoylcoprogen (**29**), were isolated from *Clonostachys compactiuscula* FKR-0021. They exhibited antimalarial activity against chloroquine-sensitive and chloroquine-resistant strains of *Plasmodium falciparum* strains, with IC_50_ values ranging from 1.7 μM to 9.9 μM [19].

Gliocladins A (**34**), B (**35**) and C (**36**) and glioperazine (**43**) were isolated from *Gliocladium* sp. originally separated from the sea hare (*Aplysia kurodai*). Gliocladin C (**36**), which was a structurally unique trioxopiperazine, showed significant cytotoxicity against the murine P388 lymphocytic leukemia cells with IC_50_ value of 2.4 μg/mL [21]. Gliocladin C (**36**) from *Gliocladium roseum* YMF1.00133 was further screened to show antinematodal activity against nematodes *Panagrellus redivivus*, *Caenothabditis elegans* and *Bursaphelenchus xylophilus* [22].

Nine epipolysulfanyldioxopiperazines isolated from *Gliocladium roseum* 1A displayed antinematodal activity against *Caenorhabditis elegans* and *Panagrellus redivivus*. The dimers, including gliocladine A (**37**), gliocladine B (**38**), sch52900 (**48**), sch52901 (**49**), verticillin A (**50**), and 11′-deoxyverticillin A (**51**) are more active than the monomers with the indole moiety, namely, gliocladines C (**39**), D (**40**) and E (**41**). Among them, 11′-Deoxyverticillin A (**51**) was the most potent antinematodal compound [23].

Three dioxopiperazines: glioperazine (**43**), glioperazine B (**44**) and glioperazine C (**45**) were isolated from *Bionectria byssicola* F120. Among them, glioperazine B (**44**) showed weak antibacterial activity against *Staphylococcus aureus* [25].

Haematocin (**46**) was isolated from the culture broth of *Nectria haematococca*, the blight disease pathogen of ornamental plants. Haematocin (**46**) inhibited the germ-tube elongation and spore germination of rice blast pathogen *Pyricularia oryzae* at the IC_50_ values of 30 and 160 μg/mL, respectively [26].

Verticillins were the dimeric epipolythiodioxopiperazines widely distributed in Bionectriaceous fungi. Most of verticillins exhibited cytotoxic activities [11,27]. Among them, verticillin A (**50**) showed obviously cytotoxic acitivity by causing apoptosis and reducing tumor burden in high-grade serious ovarian cancer by inducing DNA damage [42]. Verticillins D (**54**) and G (**55**) were isolated from *Bionectria byssicola*, and verticillin G (**55**) was screened to have antibacterial activity on *Staphylococcus aureus* with MIC values of 3–10 μg/mL [25]. Verticillin D (**54**) from the endophytic fungus *Bionectria ochroleuca* showed pronounced cytotoxic activity against mouse lymphoma cells [4].

### 2.4. Other Nitrogen-Containing Metabolites

**Figure 4:**
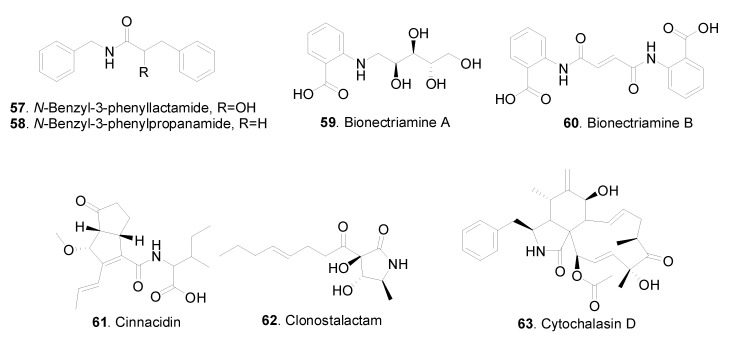

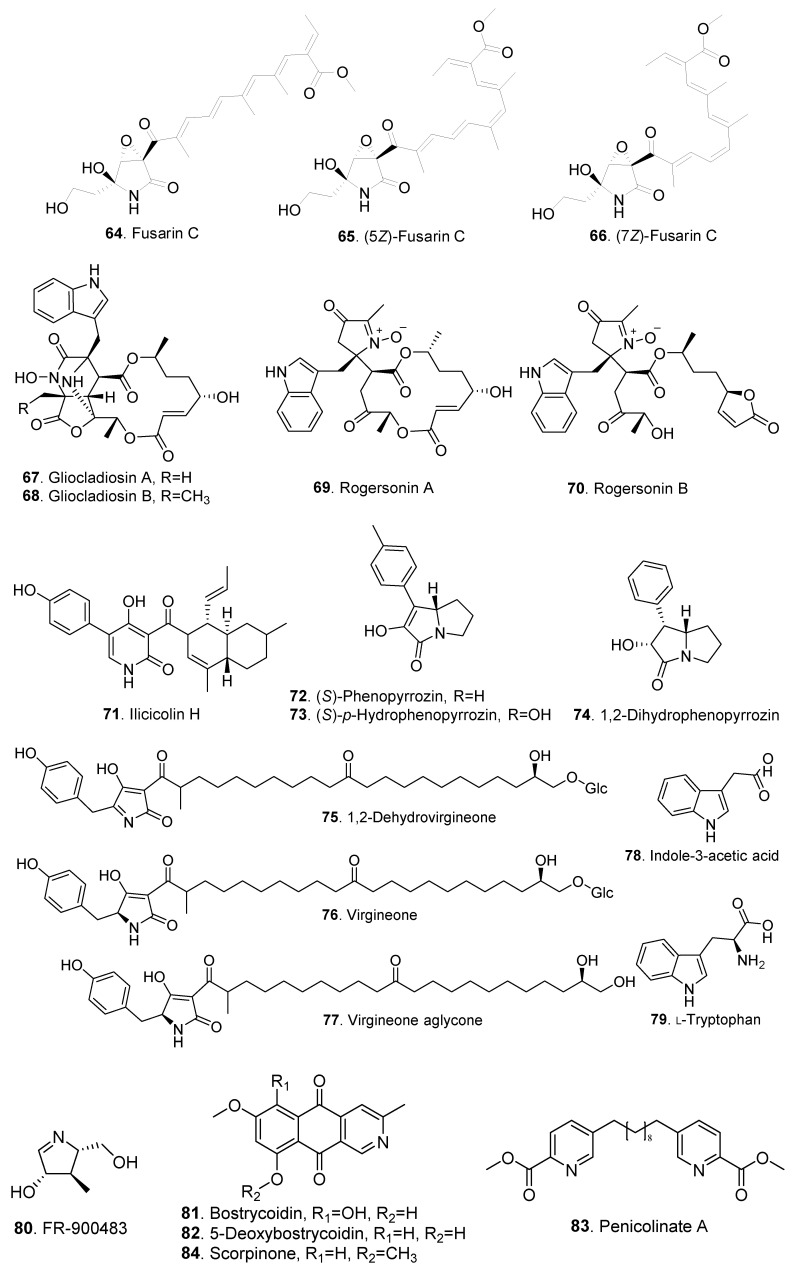
Other nitrogen-containing metabolites isolated from *Clonostachys* fungi.

The structures of the other nitrogen-containing metabolites, including amides and amines isolated from *Clonostachys* fungi are shown in Figure 4.

Both N-benzyl-3-phenyllactamide (**57**) and N-benzyl-3-phenylpropanamide (**58**) were isolated from *Clonostachys compactiuscula* FKR-0021 [19].

Fusarin C (**64**), (5Z)-fusarin C (**65**) and (7Z)-fusarin C (**66**) were isolated from Nectria coccinea A56-9. They showed antifungal activity against *Pyricularia oryzae* by inhibiting dihydroxynaphthalene-melanin biosynthesis [31].

Gliocladiosins A (**67**) and B (**68**), the dipeptides conjugated with macrolides, were isolated from an O-methyltransferase gene, *verM* disruption mutant of the *Cordycep*-colonizing fungus *Clonostachys rogersoniana*. These two compounds showed moderate antibacterial activity on *Klebsiella pneumonia* and *Bacillus subtlilis* [32]. Similarly, rogersonins A (**69**) and B (**70**) were two indole-polyketide hydrids isolated from *verG* disruption mutant of *Clonostachys rogersoniana* [33]. Blocking the biosynthesis of secondary metabolites through the disuption of the biosynthesis-related genes provide a method to activate cryptic or silent secondary metabolites in fungi.

Three tetramic acid derivatives namely 1,2-dehydrovirgineone (**75**), virgineone (**76**) and virgineone aglycone (**77**) were isolated from *Bionectria* sp. MSX 47401. They showed obviously antibacterial activity against Staphylococcus aureus and several MRSA isolates. In addition, virgineone (**76**) showed moderate antifungal activity against *Candida albicans*, *Cryptococcus neoformans*, and *Aspergillus niger* with an MIC value of 14.4 μg/mL [3].

FR-900483 (**80**), which was called nectrisine or 3-(R)-4-(R)-dihydroxy-5-(R)-hydroxymethyl-1-pyrroline, was an immunoactive substance produced by *Nectria lucida* F-4490. FR-900483 (**80**) could restore the capacity of immunosuppressed mice to produce antibody against sheep red blood cells [35].

Penicolinate A (**83**) was induced from the endophytic fungus *Bionectria* sp. through bacterial co-culture. Penicolinate A (**83**) exhibited potent cytotoxic activity against the human ovarian cancer cell line A2780 with an IC_50_ value of 4.1 μM [28].

## 3. Polyketides and Their Biological Activities

A variety of polyketides occur widely in the *Clonostachys* fungi. According to the structure characteristics, these metabolites were classifed into aromatic, alipahtic and mixed biogenic polyketides [43]. The aromatic polyketides mainly include pyranones, quinones, sorbicillinoids, and others. The polyketides, their isolated *Clonostachys* fungi and biological activities are shown in Table 2.

### 3.1. Pyranones

**Figure 5:**
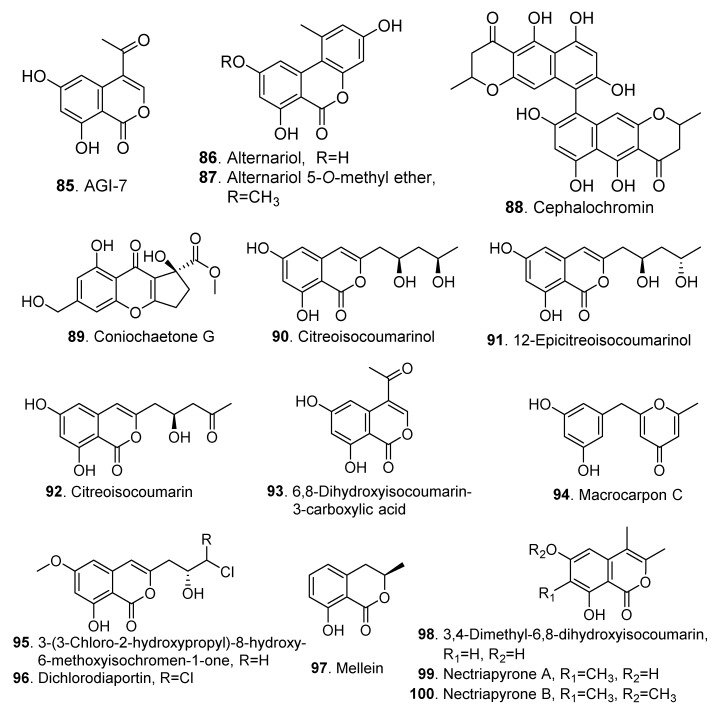

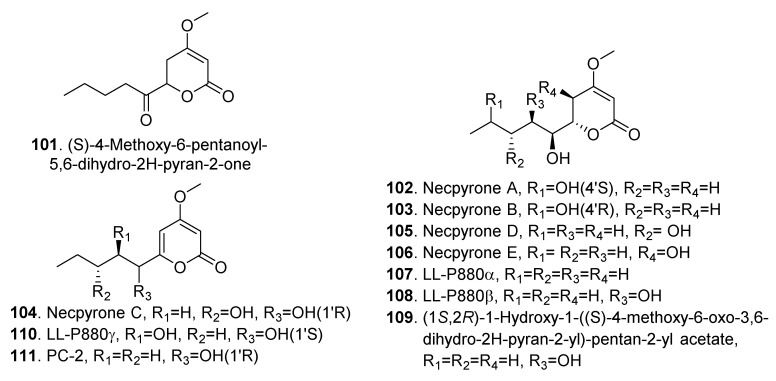
Pyranones isolated from *Clonostachys* fungi.

Pyranones (also named pyrones) from fungi include *α*-, *β*- and *γ*-pyranones [69]. Most pyranones produced by *Clonostachys* fungi belong to *α*-pyranones. Their structures are shown in Figure 5.

Cephalochromin (**88**), a bisnaphtho-γ-pyrone, was isolated from *Nectria viridescens* [45]. This compound was screened to exhibit cytotoxic activity by inducing G0/G1 cell cycle arrest and apoptosis in A549 human non-small-cell lung cancer cells by inflicting mitochondrial disruption [46].

Citreoisocoumarinol (**90**), citreoisocoumarin (**92**) and macrocarpon C (**94**) showed moderate inhibitory activity on α-glucosidase with IC_50_ values ranging from 300 to 600 µM [47].

Two isocoumarin derivatives, 3-(3-chloro-2-hydroxypropyl)-8-hydroxy- 6-methoxyisochromen-1-one (**95**) and 3-[(R)-3,3-dichloro-2-hydroxypropyl]- 8-hydroxy-6- methoxy-1*H*-isochromen-1-one (dichlorodiaportin, **96**), were identified from *Clonostachys* sp. AP4.1 [48].

### 3.2. Quinones

**Figure 6:**
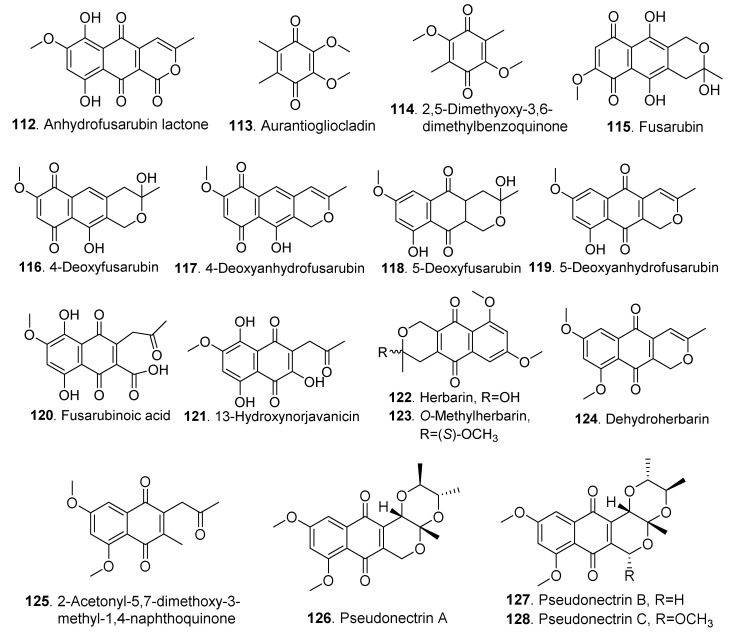

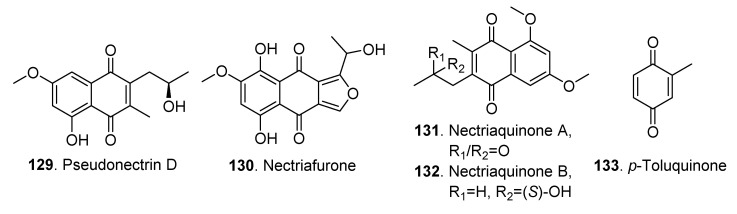
Quinones isolated from *Clonostachys* fungi.

The quinones isolated from Clonostachys fungi were mainly naphthoquinones except for three p-benzoquinones. Their structures are shown in Figure 6.

2,5-Dimethoxy-3,6-dimethyl-1,4-benzoquinone (**114**) from *Nectria coryli* inhibited the growth of *Staphylococcus aureus* at a concentration of 1 μg/mL [54].

Herbarin (**122**) and nectriaquinone B (**132**) isolated from the brown rice culture of *Nectria pseudotrichia* 120-1NP exhibited antibacterial activities against *Staphylococcus aureus* and *Pseudomonas aeruginosa* [50]. Herbarin (**122**), *O*-methylherbarin (**123**), nectriaquinone A (**131**), and nectriaquinone B (**132**) displayed cytotoxic activity against human promyelocytic leukemia HL60 cells with IC_50_ values of 11.9, 1.33, 1.93, and 11.6 μM, respectively. The structure-function relationship elucidated that the higher cytotoxicity of herbarin (**122**) and nectriaquinone B (**132**), compared to that of the related compounds *O*-methylherbarin (**123**) and nectriaquinone A (**131**) was attributed to their increased cell membrane permeability due to the presence of the hydroxyl group [38,50]. In addition, herbarin (**122**) showed a significant inhibition on lettuce seedling growth [50].

Seven naphthoquinones, named pseudonectrins A (**126**), B (**127**), C (**128**), D (**129**), herbarin (**122**), dehydroherbarin (**124**) and 2-acetoxyl-5,7-dimethoxy-3-methyl-1,4-naphthoquinone (**125**) were isolated from *Nectria pseudotrichia*. They all showed cytotoxic activity except for pseudonectrin D (**129**). In addition, pseudonectrins A (**126**), B (**127**) and C (**128**) had a skeleton of pyranonaphthoquinone [38].

### 3.3. Sorbicillinoids

**Figure 7:**
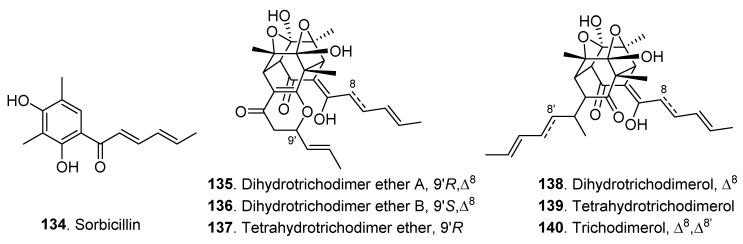
Sorbicillinoids isolated from *Clonostachys* fungi.

Sorbicillinoids are important hexaketide metabolites produced by fungi [70]. Six dimeric and one monomeric sorbicillinoids were extracted from culture broth of *Clonostachys rosea* YRS-06 [44]. Their structures are shown in Figure 7. Dihydrotrichodimer ether A (**135**), dihydrotrichodimer ether B (**136**) and tetrahydrotrichodimer ether (**137**) are rare bisorbicillinoids with a *γ*-pyrone moiety. Dihydrotrichodimer ether A (**135**), dihydrotrichodimer ether B (**136**), dihydrotrichodimerol (**138**) and tetrahydrotrichodimerol (**139**) showed antibacterial activity against *Bacillus subtilis*, *Clostridium perfringens*, and *Escherichia coli* [44].

### 3.4. Other Polyketides

**Figure 8:**
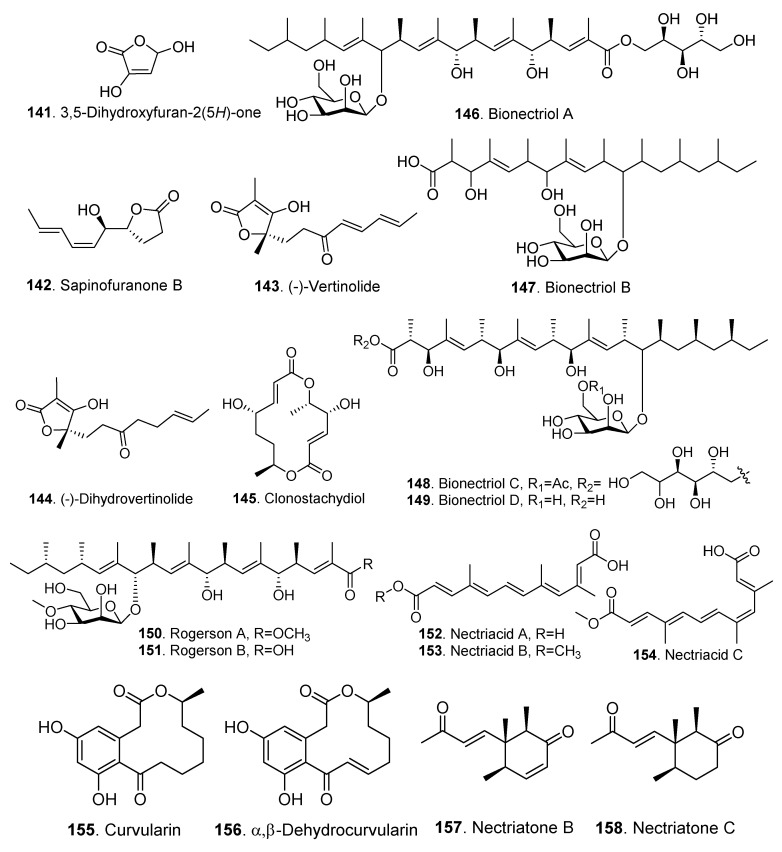

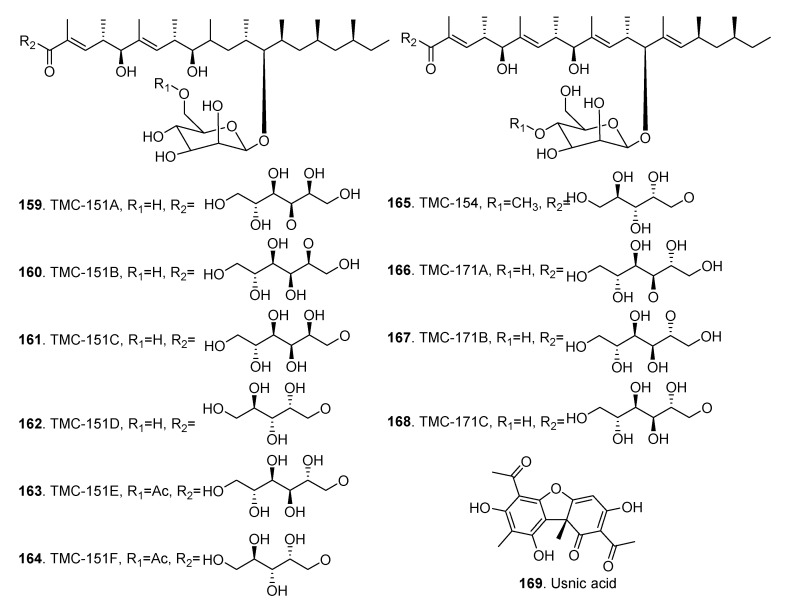
Other polyketides isolated from *Clonostachys* fungi.

The structures of the other polyketides isolated from *Clonostachys* fungi are shown in Figure 8. These metabolites mainly belong to aliphatic polyketides. Some of them contain a glycosyl group and exist as glycosides.

Four α-furanones were obtained. Both 3,5-dihydroxyfuran-2(5H)-one (**141**) and sapinofuranone B (**142**) were isolated from *Gliocladium roseum* 1A [23]. Both (-)-vertinolide (**143**) and (-)-dihydrovertinolide (**144**) were isolated from *Clonostachys rosea* B5-2. (-)-Dihydrovertinolide (**144**) displayed phytotoxic activity against lettuce seedlings at a concentration of 50 μg/mL [58].

Clonostachydiol (**145**) was a 14-membered macrodiolide isolated from the fungus *Clonostachys cylindrospora* (strain FH-A 6607). It exhibited anthelimintic activity against abomasum nematode *Haemonchus cortorus* in artificially infected lambs [59]. Four stereocenters in clonostachydiol were revised later [71].

Polyketide glycosides bionectriols A (**146**), B (**147**) and C (**148**) were isolated from *Bionectria chroleuca* [61]. TMC-151E (**163**), TMC-151F (**164**) and bionectriol C (**148**) moderately inhibited *Candida albicans* biofilm formation with IC_50_ values of 36.3, 41.0 and 24.1 μM, respectively [61].

Nectriacids B (**153**) and C (**154**) showed stronger α-glucosidase inhibitory activity than positive control (acarbose, IC_50_, 815.3 µM) with IC_50_ values of 23.5 and 42.3 µM, respectively.

*α*,*β*-Dehydrocurvularin (**156**) from *Nectria glligena* was proved to be cytotoxic to human lung fibroblasts with IC_50_ value less than 12 µg/mL. In addition, *α*,*β*-dehydrocurvularin (**156**) significantly reduced radicle length and epicotyl growth in *Lactuca sativa* at 100 and 200 µg/disk [63].

Both nectriatones B (**157**) and C (**158**) were cyclohexanone derivatives from *Nectria* sp. B-13 [64].

A series of polyketides TMC-151 (**159**–**164**), TMC-154 (**165**) and TMC-171 (**166**–**168**) were found exclusively in *Gliocladium* and *Clonostachys* species [67]. They contained _D_-mannopyranoside and _D_-mannitol or _D_-arabitol and showed moderate cytotoxicity on several tumor cells [66].

Usnic acid (**169**) is a unique polyketide from *Bionectria ochroleuca* Bo-1 which was isolated as an endophytic fungus from rice. It showed antibacterial activity against *Xanthomonas oryzae* with MIC value of 200 µg/mL [68].

## 4. Terpenoids and Their Biological Activities

The terpenoids from *Clonostachys* fungi include monoterpenoids, sesquiterpenoids, diterpenoids, triterpenpoids, polyterpenoids, and meroterpenoids. The terpenoids, along with their isolated *Clonostachys* fungi and biological activities are shown in Table 3.

### 4.1. Monoterpenoids

**Figure 9:**
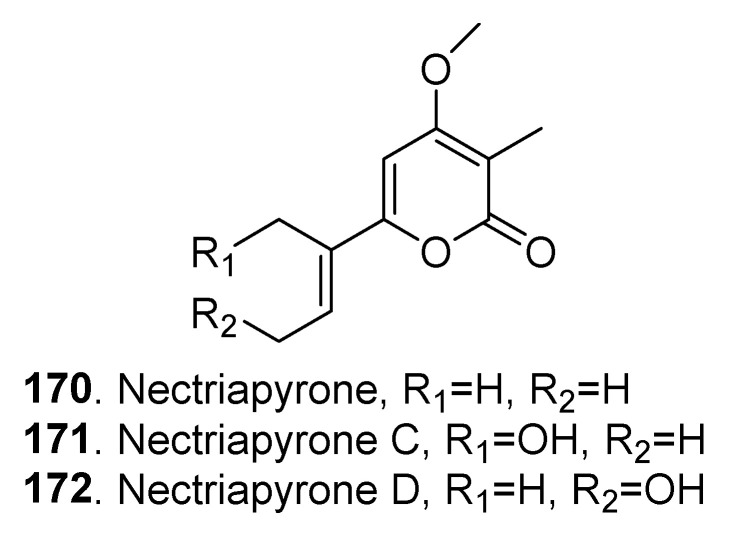
Monoterpenoids isolated from *Clonostachys* fungi.

Three monoterpenoids named nectriapyrone (**170**), nectriapyrones C (**171**) and D (**172**) with α–pyrone skeletons were isolated from the fungus *Nectria* sp. HLS206 associated with the marine sponge *Gelliodes carnosa* [72]. Their structures are shown in Figure 9.

### 4.2. Sesquiterpenoids

**Figure 10:**
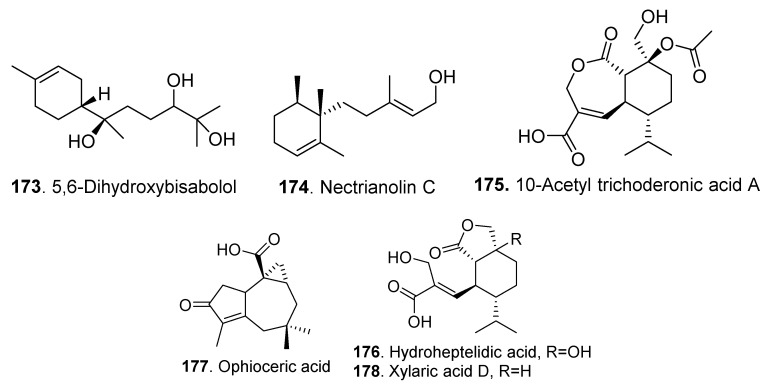
Sesquiterpenoids isolated from *Clonostachys* fungi.

The structures of the sesquiterpenoids isolated from *Clonostachys* fungi are shown in Figure 10. Nectrianolin C (**174**) from *Nectria pseudotrichia* 120-1NP exhibited cytotoxic activity against HL60 and HeLa cells [73].

Three sesquiterpene acids: 10-acetyl trichoderonic acid A (**175**), hydroheptelidic acid (**176**), and xylaric acid D (**178**) were isolated from the endophytic fungus *Nectria pseudotrichia* of the tree *Caesalpinia echinata*. The 10-Acetyl trichoderonic acid A (**175**) and hydroheptelidic acid (**176**) showed strong antileishmanial activity [30].

### 4.3. Diterpenoids

**Figure 11:**
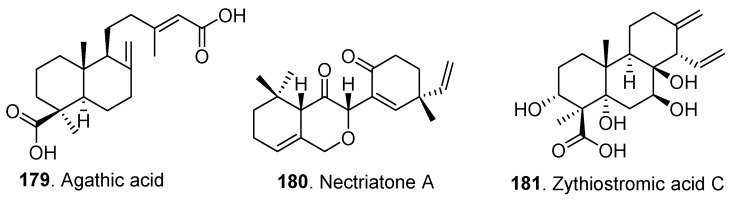
Diterpenoids isolated from *Clonostachys* fungi.

Three diterpenoids (**179**–**181**) have been isolated from *Clonostachys* fungi so far (Figure 11). Nectriatone A (**180**) from *Nectria* sp. B-13 exhibited cytotoxic activity against the human cancer cell lines, including SW1990, HCT-116, MCF-7 and K562 [64].

### 4.4. Triterpenoids

**Figure 12:**
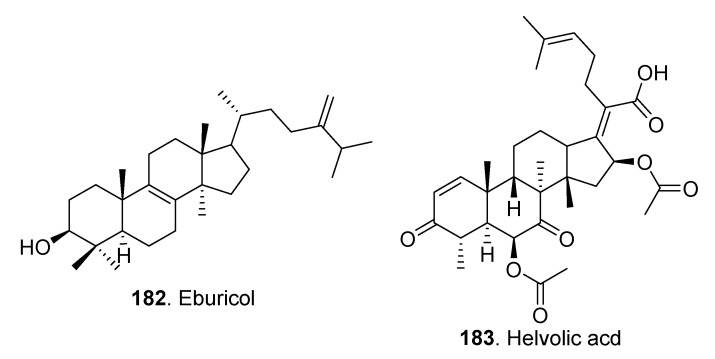
Triterpenoids isolated from *Clonostachys* fungi.

Only two triterpenoids (**182**, **183**) were described with their structures shown in Figure 12. Eburicol (**182**) exhibited cytotoxic activities on the four human cancer cell lines, which included MCF-7, MDA-MB-231, NSCLC-N6-L16 and A549 cells with IC_50_ values lower than 40 µM [74]. Helvolic acid (**183**) was a nortriterpenoid isolated from many other fungi, such as *Pichia guilliermondii* [75], and *Aspergillus fumiatus* [76]. This compound exhibited obvious antimicrobial activity [75,76].

### 4.5. Polyterpenoids

**Figure 13:**
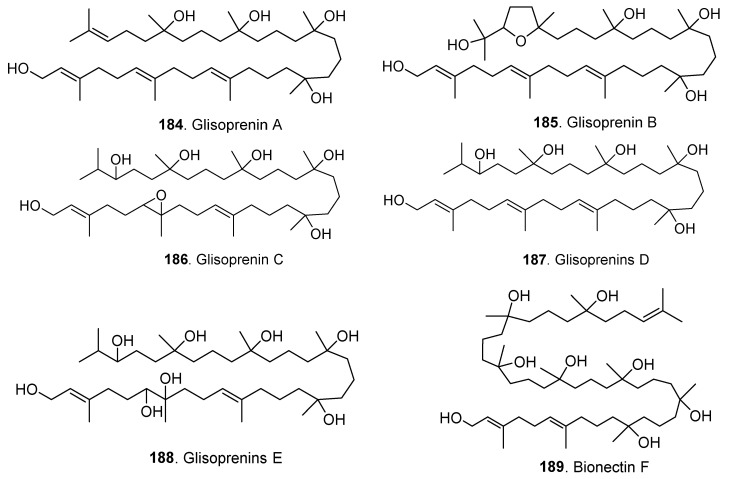
Polyterpenoids isolated from *Clonostachys* fungi.

The polyterpenes in *Clonostachys* fungi were tetraterpenes or pentaterpenes whose structures are shown in Figure 13. Five polyprenol polyterpenoids, glioprenins A–E (**184**–**188**) were isolated from *Gliocladium* species [77,78]. Glisoprenins A (**184**) and B (**185**) from *Gliocladium* sp. FO-1513 showed inhibitory activity on acyl-CoA:cholesterol acyltransferase [77], and glioprenins C (**186**), D (**187**) and E (**188**) from the submerged cultures of *Gliocladium roeum* HA190-95 showed inhibition on appressorium formation of *Magnaporthe grisea* [78].

Bionectin F (**189**), another polyprenol polyterpenoid, was isolated from the endophytic fungus *Bionectria* sp. Y1085 [17].

### 4.6. Meroterpenoids

**Figure 14:**
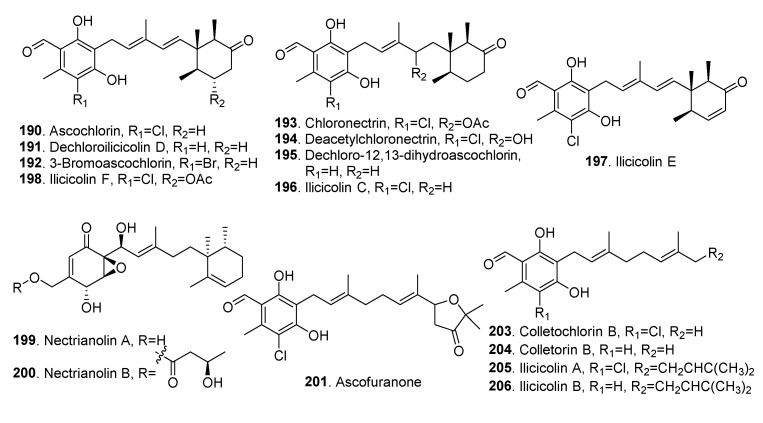

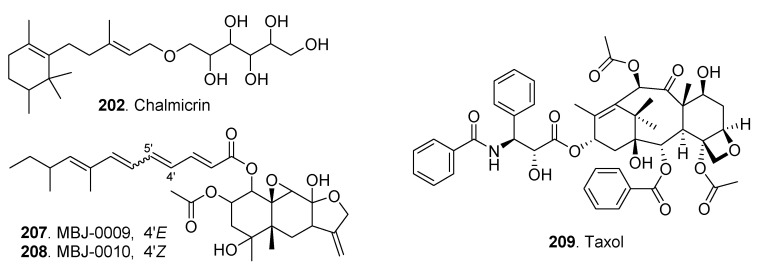
Meroterpenoids isolated from *Clonostachys* fungi.

Meroterpenoids are metabolites that are partially derived from terpenoid biosynthetic pathways. The structures of meroterpenoids isolated from *Clonostachys* fungi are shown in Figure 14.

Ascochlorin (also named illicolin D or LL-Z 1272γ, **190**), dechlorodihydroascochlorin (**195**) and ilicicolin B (or called LL-Z 1272β, **206**) were isolated from *Nectria* sp. [79].

Ilicicolins D (**190**), E (**197**) and F (**198**), dechloroilcicolin D (**191**) and ascofuranone (**201**) showed antifungal activity against plant pathogens *Neurospora crassa*, *Botrytis cinerea*, *Fusariumculmorum*, *Pyricularia oryzae*, and *Penicillium digitarum* [8].

Ilicicolins C (**196**) and E (**197**), and colletochlorin B (**203**) from the phytopathogenic fungus *Nectria galligena* displayed inhibitory activity toward acetylcholinesterase (AChE) and α-glucuronidase with IC_50_ values of 30-36 µg/mL in the AChE assay and 32-43 µg/mL in the α-glucuronidase test [63].

Ilicicolin E (**197**) obtained from soil-derived fungus Nectria sp. B-13 showed antibacterial activities against *Escherichia coli*, *Bacillus subtilis* and *Staphylococcus aureus* with MIC values of 4.0, 2.0 and 4.0 μg/mL, respectively [64]. Ilicicolin C (**196**) and ilicicolin F (**198**) obtained from phytopathogenic fungus Nectria galligena were active against *Pseudomonas syringae* with IC_50_ values of 28.5 and 28.5 μg/mL, respectively [63].

Both nectrianolins A (**199**) and B (**200**) were sesquiterpene-epoxyclohexenone conjugates isolated from *Nectria pseudotrichia* 120-1NP [73].

Chalmicrin (**202**), a mannitol ether of methylated monocyclofarnesol, was isolated from Nectria sp. HLS206 that was associated with the marine sponge *Glliodes carnosa* [81]. This compound was previously isolated from the fungus *Chalara microspora* [84].

Both MBJ-0009 (**207**) and MBJ-0010 (**208**), which were related to the eremophilane class and isolated from the saprobic fungus Nectria sp. f26111, showed moderate cytotoxic activity against human ovarian adenocarcinoma SKOV-3 with the IC_50_ values of 24.7 and 11.2 mM, respectively [82].

Taxol (generic name paclitaxel, **209**), the well-known anticancer agent, was isolated from the endophytic fungus *Gliocladium* sp. from *Taxus baccata* [83]. The backbone of taxol (**209**) is a diterpenoid, and the side chain is phenylalanine-derived. Both diterpenoid and phenylpropanoid pathways are required for taxol biosynthesis. Taxol (**209**) was found in both plants [85] and fungi [86]. It is a result of the co-evolution of plants and fungi in secondary metabolism [75].

## 5. Miscellaneous Metabolites and Their Biological Activities

**Figure 15:**
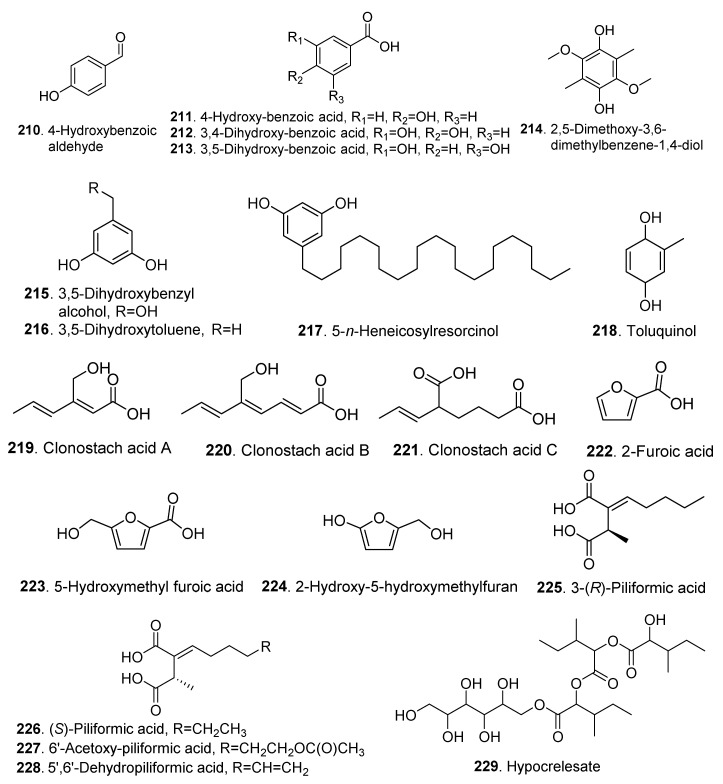
Miscellaneous metabolites isolated from *Clonostachys* fungi.

The miscellaneous metabolites mainly including phenolics and fatty acids isolated from *Clonostachys* fungi are listed in Table 4, and their structures are shown in Figure 15.

Four phenolic metabolites were isolated and identified as 4-hydroxybenzoic aldehyde (**210**), 4-hydroxybenzoic acid (**211**), 3,4-dihydroxybenzoid acid (**212**), and 3,5-dihydroxybenzoic acid (**213**) from *Gliocladium roseum* CGMCC 3.3657 [87].

5-*n*-Heneicosylresorcinol (**217**) was isolated from *Gliocladium roseum* YMF1.00133. After 24 h incubation, 5-*n*-heneicosylresorcinol (**217**) showed antinematodal activity against *Caenothabditis elegans* at 15 and 30 μg/mL, against *Panagrellus redivivus* at 50 and 80 μg/mL, and against *Bursaphelenchus xylophilus* at 200 and 180 μg/mL, respectively [22].

Five fatty acids named clonostach acids A (**219**), B (**220**), and C (**221**) were isolated from the endophytic fungus *Clonostachys rosea* B5-2 [58].

Three furan derivatives, named 2-furoic acid (**222**), 5-hydroxymethyl furoic acid (**223**) and 2-hydroxy-5-hydroxymethyl furan (**224**), were isolated from *Bionectria* sp. Y1085 [17].

Three piliformic acid derivatives were isolated from *Nectria pseutrichia*. Both 3-(S)-piliformic acid (**226**) and 6’-acetoxy-piliformic acid (**227**) were screened to show leishmanicidal activity [30].

## 6. Conclusions and Future Perspectives

In this mini-review, we summarized chemical structures, occurrences and biological activities of the secondary metabolites from *Clonostachys* fungi. The main metabolites belong to nitrogen-containing compounds, polyketides, and terpenoids. Some piperazines (i.e., bionetins, gliocladins and gliocladins and verticillins), polyketides (i.e., nectriaquinones, pesudonectrins, bionectriols, and nectriacids) and terpenoids (i.e., glisoprenins and ilicicolins), which were only isolated from *Clonostachys* fungi, exhibited obvious biological activities, such as antimicrobial, cytotoxic, antinematodal, and AChE inhibitory activities (Table 1, Table 2 and Table 3). Some metabolites, such as alternariol (**86**) sorbicillinoids, were also distributed in other groups of fungi. Some metabolites, such as cyclosporin A (**12**), cinnacidin (**61**), and taxol (**209**), have shown their medicinal and agricultural applications.

In order to search for new bioactive metabolites from *Clonostachy* fungi, some strategies, such as gene disruption, modification of the fermentation medium, co-cultivation and synthetic modification, have been proven to be effective. Gliocladiosins A (**67**) and B (**68**), as well as rogersonins A (**69**) and B (**70)** were alkaloid–polyketide hydrids isolated from gene disruption mutants of *Clonostachys rogersoniana* [32,33]. Fermentation of the *Clonostachys rosea* on white beans instead of rice afforded one *γ*-lactam clonostalactam (**62**) and two *γ-*lactones 3,5-dihydroxyfuran-2(5*H*)-one (**141**) and sapinofuranone B (**142**) that were not detected in the former extracts [11]. The apple juice supplemented solid rice media led to significant changes in the secondary metabolism of the fungus, *Clonostachys rosea* B5-2, and induced the production of four new compounds, (-)-dihydrovertinolide (**144**), clonostach acids A (**219**), B (**220**) and C (**221**) together with the known compound, (-)-vertinolide (**143**) [58]. Co-cultivation of the *Bionectria* sp. with either *Bacillus subtilis* or *Streptomyces lividans* resulted in the production of bionectriamines A (**59**) and B (**60**), and 6,8-dihydroxyisocoumarin-3-carboxylic acid (**93**) [28]. In addition, based on the isolated compounds, more bioactive compounds can be synthesized. A typical example was the synthesis of cinnacidin (**61**) analogs. Two new structural analogs of cinnacidin (**61**), namely (2*S*,3*S*)-2-[(3*RS*,3a*SR*,6a*RS*)-3-methoxy-4-oxo-3,3a,4,5,6,6a-hexahydropentalen-1-ylcarbamoyl]-3-methylvaleric acid and benzyl (2*S*,3*S*)-2-[(3*RS*,3a*SR*,6a*RS*)-3-methoxy-4-oxo-3,3a,4, 5,6,6a-hexahydro pentalen-1-ylcarbamoyl]-3-methylvalerate, have been synthesized. The synthetic compounds were highly phytotoxic on a range of weeds to show their potential application as an herbicide [29]. Furthermore, the phenolic sesquiterpenoids which are also called ascochlorin derivatives or ilicicolins were widely distributed in the fungi of genus *Nectria* (synonym: *Clonostachys*). The occurrence of these compounds further confirms the close chemotaxonomic relationships among the related *Nectria* species. Ilicicolin H (**71**) was considered to be of potential chemotaxonomic significance and could be used as the main chemotaxonomic marker of the Nectriaceae family [34]. Some piperazines, such as bionetins [16], gliocladins [21], gliocladines [23], glioperazines, and verticillins were also only isolated from the fungal species of *Clonostachys*. Their chemotaxonomic significance should be further verified.

Though major fungal species of *Clonostachys* fungi have been studied for their metabolites [1], the remaining fungi need to be revealed in detail. Moreover, the biological activities, structure–activity relationships, mechanisms of action, as well as biosynthesis of the metabolites from *Clonostachys* fungi need to be further investigated. Clarification of the metabolites of *Clonostachys* fungi could not only be in favor of discovering more compounds with novel structures and excellent biological activities, but also better understand the chemotaxonomy of the genus *Clonostachys*.

## Figures and Tables

**Table 1:** Nitrogen-containing metabolites in *Clonostachys* fungi and their biological activities.

Metabolite Class	Metabolite Name	Fungal Species	Biological Activity	Ref.
Linear oligopeptides	Clonostachin (**1**)	*Clonostachys* sp. F5898	Inhibition on platelet aggregation	[2]
		*Bionectria* sp. MSX 47401	-	[3]
	Clonostachin B (**2**)	*Bionectria* sp. MSX 47401	-	[3]
	Pullularin F (**3**)	*Bionectria ochroleuca*	-	[4]
Cyclopeptides	Argadin (**4**)	*Clonostachys* sp. FO-7314	Inhibitory activity on chitinase	[5]
	Argifin (**5**)	*Gliocladium* sp. FTD-0668	Inhibitory activity on chitinase	[6,7]
	Arthrichitin (**6**)	*Nectria* sp.	-	[8]
			Antifungal activity on the yeasts	[9]
	Clonostachysin A (**7**)	*Clonostachys rogersoniana*	Anti-dinoflagellate activity	[10]
	Clonostachysin B **(8**)	*Clonostachys rogersoniana*	Anti-dinoflagellate activity	[10]
	Cyclo-(Gly-_D_-Leu-_D_-*allo*-Ile-_L_-Val-_L_-Val-_D_-Trp-β-Ala) (**9**)	*Clonostachys rosea*	Cytotoxic activity	[11]
	Cyclo-(Gly-_D_-Leu-_L_-Val-_L_-Val-_L_-Val-_D_-Trp-β-Ala) (**10**)	*Clonostachys rosea*	-	[11]
	Cyclo-(Gly-_D_-Leu-_D_-allo-Ile-_D_-*allo*-Ile-_L_-Val-_D_-Trp-β-Ala) (**11**)	*Clonostachys rosea*	-	[11]
	Cyclosporin A (**12**)	*Nectria* sp. F-4908	-	[12]
			Immunosuppressive and antifungal activities	[13]
	Cyclosporin C (**13**)	*Nectria* sp. F-4908	-	[12]
			Immunosuppressive and antifungal activities	[13]
	IB-01212 (**14**)	*Clonostachys* sp. ESNA-A009	Cytotoxic activity	[14]
			Antileishmanial activity	[15]
	Pullularin A (**15**)	*Bionectria ochroleuca*	Cytotoxic activity	[4]
	Pullularin C (**16**)	*Bionectria ochroleuca*	Cytotoxic activity	[4]
	Pullularin E (**17**)	*Bionectria ochroleuca*	-	[4]
Piperazines	Bionectin A (**18**)	*Bionectria byssicola* F120	Antibacterial activity	[16]
	Bionectin B (**19**)	*Bionectria byssicola* F120	Antibacterial activity	[16]
	Bionectin C (**20**)	*Bionectria byssicola* F120	-	[16]
	Bionectin D (**21**)	*Bionectria* sp. Y1085	Antibacterial activity	[17]
	Bionectin E (**22**)	*Bionectria* sp. Y1085	Antibacterial activity	[17]
	3,6-Bis(methylthio)-cyclo(alanyltryptophyl) (**23**)	*Nectria inventa*	Trypanocidal activity	[18]
	Chaetocin (**24**)	*Nectria inventa*	Trypanocidal activity	[18]
	Chetoseminudin B (**25**)	*Nectria inventa*	Trypanocidal activity	[18]
	Clonocoprogen A (**26**)	*Clonostachys compactiuscula* FKR-0021	Antimalarial activity	[19]
	Clonocoprogen B (**27**)	*Clonostachys compactiuscula* FKR-0021	Antimalarial activity	[19]
	Clonocoprogen C (**28**)	*Clonostachys compactiuscula* FKR-0021	Antimalarial activity	[19]
	*N*^14^-Plmitoylcoprogen (**29**)	*Clonostachys compactiuscula* FKR-0021	Antimalarial activity	[19]
	Cyclo (_L_-Pro-_L_-Leu) (**30**)	*Bionectria* sp. Y1085	-	[17]
	Dioxopiperazine (**31**)	*Bionectria* sp. Y1085	-	[17]
	Gliocladicillin A (**32**)	*Bionectria* sp. Y1085	-	[17]
	Gliocladicillin C (**33**)	*Bionectria* sp. Y1085	Antibacterial activity	[17]
		*Clonostachys rogersoniana*	Cytotoxic activity	[20]
	Gliocladin A (**34**)	*Glicladium roseum* OUPS-N132	-	[21]
	Gliocladin B (**35**)	*Glicladium roseum* OUPS-N132	-	[21]
	Gliocladin C (**36**)	*Glicladium roseum* OUPS-N132	Cytotoxic activity	[21]
		*Gliocladium roseum* YMF1.00133	Antinematodal activity	[22]
	Gliocladine A (**37**)	*Gliocladium roseum* 1A	Antinematodal activity	[23]
	Gliocladine B (**38**)	*Gliocladium roseum* YMF1.00133	Antinematodal activity	[23]
		*Bionectria* sp. Y1085	-	[17]
	Gliocladine C (**39**)	*Gliocladium roseum* 1A	Antinematodal activity	[23]
	Gliocladine D (**40**)	*Gliocladium roseum* 1A	Antinematodal activity	[23]
	Gliocladine E (**41**)	*Gliocladium roseum* 1A	Antinematodal activity	[23]
	Glioclatine (**42**)	*Gliocladium roseum* YMF1.00133	Antinematodal activity	[24]
	Glioperazine (**43**)	*Gliocladium* sp. OUPS-N132	-	[21]
		*Bionectria byssicola* F120	-	[25]
		*Clonostachys rosea*	-	[11]
	Glioperazine B (**44**)	*Bionectria byssicola* F120	Antimicrobial activity	[25]
	Glioperazine C (**45**)	*Bionectria byssicola* F120	-	[25]
	Haematocin (**46**)	*Nectria haematococca*	Antifuangl activity	[26]
	Lasiodipline D (**47**)	*Bionectria* sp. Y1085	-	[17]
	Sch52900 (**48**)	*Gliocladium roseum* 1A	Antinematodal activity	[23]
	Sch52901 (**49**)	*Gliocladium roseum* 1A	Antinematodal activity	[23]
	Verticillin A (**50**)	*Gliocladium roseum* 1A	Antinematodal activity	[23]
		*Bionectria* sp. Y1085	-	[17]
	11′-Deoxyverticillin A (**51**)	*Gliocladium roseum* 1A	Antinematodal activity	[23]
	11,11′-Dideoxyverticillin A (**52**)	*Bionectria* sp. Y1085	-	[17]
	Verticillin B (**53**)	*Nectria inventa*	Trypanocidal activity	[18]
	Verticillin D (**54**)	*Bionectria byssicola* F120	-	[16]
		*Bionectria ochroleuca*	Cytotoxic activity	[4]
		*Clonostachys rosea*	Cytotoxic activity	[11]
	Verticillin G (**55**)	*Bionectria byssicola* F120	Antibacterial activity	[25]
	Verticillin H (**56**)	Bionectriaceous strains MSX 64546 and MSX 59553	Cytotoxic activity	[27]
Other nitrogen-containing metabolites	*N*-Benzyl-3-phenyllactamide (**57**)	*Clonostachys compactiuscula* FKR-0021	-	[19]
	*N*-Benzyl-3-phenylpropanamide (**58**)	*Clonostachys compactiuscula* FKR-0021	-	[19]
	Bionectriamine A (**59**)	Co-cultivation of *Bionectria* sp. with *Bacillus subtilis* or *Streptomyceslividans*	-	[28]
	Bionectriamine B (**60**)	Co-cultivation of *Bionectria* sp. with *Bacillus subtilis* or *Streptomyceslividans*	-	[28]
	Cinnacidin (**61**)	*Nectria* sp. DA060097	Phytotoxic activity	[29]
	Clonostalactam (**62**)	*Clonostachys rosea*	-	[11]
	Cytochalasin D (**63**)	*Nectria pseudotrichia*	Weak leishmanicidal activity	[30]
	Fusarin C (**64**)	*Nectria coccinea* A56-9	Antifungal activity	[31]
	(5*Z*)-Fusarin C (**65**)	*Nectria coccinea* A56-9	Antifungal activity	[31]
	(7*Z*)-Fusarin C (**66**)	*Nectria coccinea* A56-9	Antifungal activity	[31]
	Gliocladiosin A (**67**)	*verM* disruption mutant of *Clonostachys rogersoniana*	Antibacterial activity	[32]
	Gliocladiosin B (**68**)	*verM* disruption mutant of *Clonostachys rogersoniana*	Antibacterial activity	[32]
	Rogersonin A (**69**)	*verG* disruption mutant of *Clonostachys rogersoniana*	-	[33]
	Rogersonin B (**70**)	*verG* disruption mutant of *Clonostachys rogersoniana*	-	[33]
	Ilicicolin H (**71**)	*Nectria* sp. B-13	-	[34]
	(*S*)-Phenopyrrozin (**72**)	*Bionectria* sp.	-	[28]
	(*S*)-*p*-Hydroxyphenopyrrozin (**73**)	*Bionectria* sp.	-	[28]
	1,2-Dihydrophenopyrrozin (**74**)	*Bionectria* sp.	-	[28]
	1,2-Dehydrovirgineone (**75**)	*Bionectria* sp. MSX 47401	Antibacterial activity	[3]
	Virgineone (**76**)	*Bionectria* sp. MSX 47401	Antibacterial and antifungal activities	[3]
	Virgineone aglycone (**77**)	*Bionectria* sp. MSX 47401	Antibacterial activity	[3]
	Indole-3-acetic acid (**78**)	*Bionectria* sp. Y1085	-	[17]
	_L_-Tryptophan (**79**)	*Bionectria* sp. Y1085	-	[17]
	FR-900483 (**80**)	*Nctria lucida* F-4490	Immunostimulatory activity	[35]
	Bostrycoidin (**81**)	*Nectria haematococca*	-	[36]
	5-Deoxybostrycoidin (**82**)	*Nectria haematococca*	-	[37]
	Penicolinate A (**83**)	*Bionectria* sp.	Cytotoxic activity	[28]
	Scorpinone (**84**)	*Nectria pseudotrichia*	-	[38]

**Table 2:** Polyketides in *Clonostachys* fungi and their biological activities.

Metabolite Class	Metabolite Name	Fungal Species	Biological Activity	Ref.
Pyranones	AGI-7 (**85**)	*Bionectria* sp. MSX 47401	-	[3]
	Alternariol (**86**)	*Clonostachys rosea* YRS-06	-	[44]
	Alternariol 5-*O*-methyl ether (**87**)	*Clonostachys rosea* YRS-06	-	[44]
	Cephalochromin (**88**)	*Nectria viridescens*	-	[45]
			Cytotoxic activity	[46]
	Coniochaetone G (**89**)	*Clonostachys compactiuscula* FKR-0021	-	[19]
	Citreoisocoumarinol (**90**)	*Nectria* sp. HN001	Inhibitory activity on α-glucosidase	[47]
	12-Epicitreoisocoumarinol (**91**)	*Nectria* sp. HN001	-	[47]
	Citreoisocoumarin (**92**)	*Nectria* sp. HN001	Inhibitory activity on α-glucosidase	[47]
	6,8-Dihydroxyisocoumarin -3-carboxylic acid (**93**)	Co-cultivation of *Bionectria* sp. with *Bacillus subtilis* or *Streptomyceslividans*	-	[28]
	Macrocarpon C (**94**)	*Nectria* sp. HN001	Inhibitory activity on α-glucosidase	[47]
	3-(3-Chloro-2-hydroxypropyl)-8-hydroxy-6-methoxyisochromen-1-one (**95**)	*Clonostachys* sp. AP4.1	-	[48]
	Dichlorodiaportin (**96**)	*Clonostachys* sp. AP4.1	-	[48]
	Mellein (**97**)	*Nectria fuckeliana*	-	[49]
	3,4-Dimethyl-6,8-dihydroxyisocoumarin (**98**)	*Nectria pseudotrichia* 120-1NP	-	[50]
	Nectriapyrone A (**99**)	*Nectria pseudotrichia* 120-1NP	-	[50]
	Nectriapyrone B (**100**)	*Nectria pseudotrichia* 120-1NP	-	[50]
	(*S*)-4-Methoxy-6-pentanoyl-5,6-dihydro-2*H*-pyran-2-one (**101**)	*Nectria* sp.	-	[51]
	Necpyrone A (**102**)	*Nectria* sp.	-	[51]
	Necpyrone B (**103**)	*Nectria* sp.	-	[51]
	Necpyrone C (**104**)	*Nectria* sp.	-	[51]
	Necpyrone D (**105**)	*Nectria* sp.	-	[51]
	Necpyrone E (**106**)	*Nectria* sp.	-	[51]
	LL-P880α (**107**)	*Nectria* sp.	-	[51]
	LL-P880β (**108**)	*Nectria* sp.	-	[51]
	(1*S*, 2*R*)-1-Hydroxy-1-((*S*)-4-methoxy-6-oxo-3,6-dihydro-2*H*-pyran-2-yl)-pentan-2-yl acetate (**109**)	*Nectria* sp.	-	[51]
	LL-P880γ (**110**)	*Nectria* sp.	-	[51]
	PC-2 (**111**)	*Nectria* sp.	-	[51]
Quinones	Anhydrofusarubin lactone (**112**)	*Nectria haematococca*	-	[52]
	Aurantiogliocladin (**113**)	*Clonostachys candelabrum*	-	[53]
	2,5-Dimethyoxy-3,6-dimethyl-1,4-benzoquinone (**114**)	*Nectria coryli*	Antibacterial activity	[54]
		*Nectria fuckeliana*	-	[49]
	Fusarubin (**115**)	*Nectria haematococca*	-	[36]
	4-Deoxyfusarubin (**116**)	*Nectria haematococca*	-	[55]
	4-Deoxyanhydrofusarubin (**117**)	*Nectria haematococca*	-	[55]
	5-Deoxyfusarubin (**118**)	*Nectria haematococca*	-	[55]
	5-Deoxyanhydrofusarubin (**119**)	*Nectri haematococca*	-	[55]
	Fusarubinoic acid (**120**)	*Nectria haematococca*	-	[56]
	13-Hydroxynorjavanicin (**121**)	*Nectria haematococca*	-	[56]
	Herbarin (**122**)	*Nectria pseudotrichia* 120-1NP	Antibacterial and phytotoxic activities	[50]
		*Nectria pseudotrichia*	Cytotoxic activity	[38]
	*O*-Methylherbarin (**123**)	*Nectria pseudotrichia* 120-1NP	Cytotoxic activity	[50]
	Dehydroherbarin (**124**)	*Nectria pseudotrichia*	Cytotoxi activity	[38]
	2-Acetoxyl-5,7-dimethoxy-3-methyl-1,4-naphthoquinone (**125**)	*Nectria pseudotrichia*	Cytotoxic activity	[38]
	Pseudonectrin A (**126**)	*Nectria pseudotrichia*	Cytotoxic activity	[38]
	Pseudonectrin B (**127**)	*Nectria pseudotrichia*	Cytotoxic activity	[38]
	Pseudonectrin C (**128**)	*Nectria pseudotrichia*	Cytotoxic activity	[38]
	Pseudonectrin D (**129**)	*Nectria pseudotrichia*	-	[38]
	Nectriafurone (**130**)	*Nectria haematococca*	-	[52]
	Nectriaquinone A (**131**)	*Nectria pseudotrichia* 120-1NP	Cytotoxic activity	[50]
	Nectriaquinone B (**132**)	*Nectria pseudotrichia* 120-1NP	Antibacterial and cytotoxic activities	[50]
	*P*-Toluquinone (**133**)	*Nectria erubescens*	-	[57]
Sorbicillinoids	Sorbicillin (**134**)	*Clonostachys rosea* YRS-06	-	[44]
	Dihydrotrichodimer ether A (**135**)	*Clonostachys rosea* YRS-06	Antibacterial activity	[44]
	Dihydrotrichodimer ether B (**136**)	*Clonostachys rosea* YRS-06	Antibacterial activity	[44]
	Tetrahydrotrichodimer ether (**137**)	*Clonostachys rosea* YRS-06	-	[44]
	Dihydrotrichodimerol (**138**)	*Clonostachys rosea* YRS-06	Antibacterial activity	[44]
	Tetrahydrotrichodimerol (**139**)	*Clonostachys rosea* YRS-06	Antibacterial activity	[44]
	Trichodimerol (**140**)	*Clonostachys rosea* YRS-06	-	[44]
Other polyketides	3,5-Dihydroxyfuran-2(5*H*)-one (**141**)	*Gliocladium roseum* 1A	-	[23]
		*Clonostachys rosea*	-	[11]
	Sapinofuranone B (**142**)	*Gliocladium roseum* 1A	-	[23]
		*Clonostachys rosea*	-	[11]
	(-)-Vertinolide (**143**)	*Clonostachys rosea* B5-2	-	[58]
	(-)-Dihydrovertinolide (**144**)	*Clonostachys rosea* B5-2	Phytotoxic activity	[58]
	Clonostachydiol (**145**)	*Clonostachys cylindrospora* FH-A 6607	Anthelmintic activity	[59]
	Bionectriol A (**146**)	*Bionectria* sp.	-	[60]
	Bionectriol B (**147**)	*Bionectria ochroleuca*	-	[61]
	Bionectriol C (**148**)	*Bionectria ochroleuca*	Antifungal activity	[61]
	Bionectriol D (**149**)	*Bionectria ochroleuca*	-	[61]
	Rogerson A (**150**)	*Clonostachys rogersoniana*	-	[62]
	Rogerson B (**151**)	*Clonostachys rogersoniana*	-	[62]
	Nectriacid A (**152**)	*Nectria* sp. HN001	-	[47]
	Nectriacid B (**153**)	*Nectria* sp. HN001	Inhibitory activity on α-glucosidase	[47]
	Nectriacid C (**154**)	*Nectria* sp. HN001	Inhibitory activity on α-glucosidase	[47]
	Curvularin (**155**)	*Clonostachys compactiuscula* FKR-0021	-	[19]
	α,β-Dehydrocurvularin (**156**)	*Nectria glligena*	Cytotoxic and phytotoxic activities	[63]
	Nectriatone B (**157**)	*Nectria* sp. B-13	-	[64]
	Nectriatone C (**158**)	*Nectria* sp. B-13	-	[64]
	TMC-151A (**159**)	*Clonostachys rosea*	-	[65]
		*Gliocladium catenulatum*	Moderate cytotoxicity to tumor cells	[66]
		*Bionectria ochroleuca*	-	[67]
	TMC-151B (**160**)	*Clonostachys rosea*	-	[67]
		*Gliocladium catenulatum*	Moderate cytotoxicity to tumor cells	[66]
	TMC-151C (**161**)	*Clonostachys rosea*	-	[67]
		*Gliocladium catenulatum*	Moderate cytotoxicity to tumor cells	[66]
	TMC-151D (**162**)	*Clonostachys rosea*	-	[67]
		*Gliocladium catenulatum*	Moderate cytotoxicity to tumor cells	[66]
	TMC-151E (**163**)	*Clonostachys rosea*	-	[67]
		*Gliocladium catenulatum*	Moderate cytotoxicity to tumor cells	[66]
		*Bionectria ochroleuca*	Antifungal activity	[61]
	TMC-151F (**164**)	*Clonostachys rosea*	-	[67]
		*Gliocladium catenulatum*	Moderate cytotoxicity to tumor cells	[66]
		*Bionectria ochroleuca*	Antifungal activity	[61]
	TMC-154 (**165**)	*Gliocladium roseum*	-	[67]
	TMC-171A (**166**)	*Gliocladium roseum*	-	[67]
	TMC-171B (**167**)	*Gliocladium roseum*	-	[67]
	TMC-171C (**168**)	*Gliocladium roseum*	-	[67]
	Usnic acid (**169**)	*Bionectria ochroleuca* Bo-1	Antibacterial activity	[68]

**Table 3:** Terpenoids in *Clonostachys* fungi and their biological activities.

Metabolite Class	Metabolite Name	Fungal Species	Biological Activity	Ref.
Monoterpenoids	Nectriapyrone (**170**)	*Nectria* sp. HLS206	-	[72]
	Nectriapyrone C (**171**)	*Nectria* sp. HLS206	-	[72]
	Nectriapyrone D (**172**)	*Nectria* sp. HLS206	-	[72]
Sesquiterpenoids	5,6-Dihydroxybisabolol (**173**)	*Bionectria* sp. MSX 47401	-	[3]
	Nectrianolin C (**174**)	*Nectria pseudotrichia* 120-1NP	Cytotoxic activity	[73]
	10-Acetyl trichoderonic acid A (**175**)	*Nectria pseudotrichia*	Leishmanicidal activity	[30]
	Hydroheptelidic acid (**176**)	*Nectria pseudotrichia*	Leishmanicidal activity	[30]
	Ophioceric acid (**177**)	*Clonostachys compactiuscula* FKR-0021	-	[19]
	Xylaric acid D (**178**)	*Nectria pseudotrichia*	-	[30]
Diterpenoids	Agathic acid (**179**)	*Bionectria* sp.	-	[28]
	Nectriatone A (**180**)	*Nectria* sp. B-13	Cytotoxic activity	[64]
	Zythiostromic acid C (**181**)	*Nectria pseudotrichia* 120-1NP	-	[50]
Triterpentoids	Eburicol (**182**)	*Clonostachys rosea* MMS1090	Cytotoxic activity	[74]
	Helvolic acid (**183**)	*Nectria* sp.	-	[51]
			Antimicrobial activity	[75,76]
Polyterpenoids	Glisoprenin A (**184**)	*Gliocladium* sp. FO-1513	Inhibition on acyl-CoA:cholesterol acyltransferase	[77]
	Glisoprenin B (**185**)	*Gliocladium* sp. FO-1513	Inhibition on acyl-CoA:cholesterol acyltransferase	[77]
	Glisoprenin C (**186**)	*Gliocladium roseum* HA190-95	Inhibition on appressorium formation of *Magnaporthe grisea*	[78]
	Glisoprenin D (**187**)	*Gliocladium roseum* HA190-95	Inhibition on appressorium formation of *Magnaporthe grisea*	[78]
	Glisoprenin E (**188**)	*Gliocladium roseum* HA190-95	Inhibition on appressorium formation of *Magnaporthe grisea*	[78]
	Bionectin F (**189**)	*Bionectria* sp. Y1085	-	[17]
Meroterpenoids	Ascochlorin = Ilicicolin D = LL-Z 1272γ (**190**)	*Nectria lucida*	-	[79]
		*Nectria* sp.	Antifungal activity	[8]
		*Nectria* sp. B-13	-	[34]
		*Nectria* sp. B-13	Cytotoxic activity	[64]
	Dechloroilicicolin D = Cylindrol (**191**)	*Nectria* sp.	Antifungal activity	[8]
	3-Bromoascochlorin (**192**)	*Nectria coccinea*	-	[80]
	Chloronectrin (**193**)	*Nectria coccinea*	-	[80]
	Deacetylchloronectrin (**194**)	*Nectria* sp. B-13	-	[34]
	Dechlorodihydroascochlorin = Dechloro-12,13-dihydroascochlorin = LL-Z 1272ε (**195**)	*Nectria lucida*	-	[79]
		*Nectria* sp. B-13	-	[34]
	Ilicicolin C = LL-Z 1272δ (**196**)	*Nectria* sp. B-13	-	[34]
		*Nectria galligena*	Inhibitory activity on AChE and α-glucuronidase	[63]
	Ilicicolin E = 8′,9′-Dehydroascochorin = Cylindrochlorin (**197**)	*Nectria* sp. B-13	Antibacterial activity	[34]
		*Nectria* sp.	Antifungal activity	[8]
		*Nectria* sp. B-13	Cytotoxic and antibacterial activities	[64]
		*Nectria galligena*	Inhibitory activity on AChE and α-glucuronidase	[63]
	Ilicicolin F = LL-Z 1272ζ (**198**)	*Nectria* sp. B-13	-	[34]
		*Nectria galligena*	-	[63]
		*Nectria* sp.	Antifungal activity	[8]
	Nectrianolin A (**199**)	*Nectria pseudotrichia* 120-1NP	Cytotoxic activity against HL60 and HeLa cells	[73]
	Nectrianolin B (**200**)	*Nectria pseudotrichia* 120-1NP	Cytotoxic activity against HL60 and HeLa cells	[73]
	Ascofuranone (**201**)	*Nectria* sp.	Antifungal activity	[8]
	Chalmicrin (**202**)	*Nectria* sp. HLS206	-	[81]
	Colletochlorin B (**203**)	*Nectria* sp. B-13	-	[34]
		*Nectria galligena*	Inhibitory activity on AChE and α-glucuronidase	[63]
	Colletorin B (**204**)	*Nectria galligena*	-	[63]
	Ilicicolin A (**205**)	*Nectria* sp. B-13	-	[34]
	Ilicicolin B = LL-Z 1272β (**206**)	*Nectria coccinea*	-	[80]
		*Nectria lucida*	-	[79]
	MBJ-0009 (**207**)	*Nectria* sp. f26111	Cytotoxic activity	[82]
	MBJ-0010 (**208**)	*Nectria* sp. f26111	Cytotoxic activity	[82]
	Taxol = Paclitaxel (**209**)	*Gliocladium* sp.	Cytotoxicity on cancer cells	[83]

**Table 4:** Miscellaneous metabolites in *Clonostachys* fungi and their biological activities.

Metabolite Name	Fungal Species	Biological Activity	Ref.
4-Hydroxybenzoic aldehyde (**210**)	*Gliocladium roseum* CGMCC 3.3657	-	[87]
4-Hydroxy-benzoic acid (**211**)	*Gliocladium roseum* CGMCC 3.3657	-	[87]
3,4-Dihydroxy-benzoic acid (**212**)	*Gliocladium roseum* CGMCC 3.3657	-	[87]
3,5-Dihydroxy-benzoic acid (**213**)	*Gliocladium roseum* CGMCC 3.3657	-	[87]
2,5-Dimethoxy-3,6-dimethylbenzene-1,4-diol (**214**)	*Nectria coryli*	-	[54]
3,5-Dihydroxybenzyl alcohol (**215**)	*Nectria* sp. B-13	-	[34]
3,5-Dihydroxytoluene (**216**)	*Nectria* sp. B-13	-	[34]
5-*n*-Heneicosylresorcinol (**217**)	*Gliocladium roseum* YMF1.00133	Antinematodal activity	[22]
Toluquinol (**218**)	*Nectria erubescens*	-	[57]
Clonostach acid A (**219**)	*Clonostachys rosea* B5-2	-	[58]
Clonostach acid B (**220**)	*Clonostachys rosea* B5-2	-	[58]
Clonostach acid C (**221**)	*Clonostachys rosea* B5-2	-	[58]
2-Furoic acid (**222**)	*Bionectria* sp. Y1085	-	[17]
5-Hydroxymethyl furoic acid (**223**)	*Bionectria* sp. Y1085	-	[17]
2-Hydroxy-5-hydroxymethyl furan (**224**)	*Bionectria* sp. Y1085	-	[17]
3-(*R*)-Piliformic acid (**225**)	*Bionectria* sp.	-	[28]
3-(*S*)-Piliformic acid (**226**)	*Nectria pseudotrichia*	Leishmanicidal activity	[30]
6′-Acetoxy-piliformic acid (**227**)	*Nectria pseudotrichia*	Leishmanicidal activity	[30]
5′,6′-Dehydropiliformic acid (**228**)	*Nectria pseudotrichia*	-	[30]
Hypocrealesate (**229**)	*Nectria* sp. HLS206	-	[81]
